# Osteoporosis therapies and coronary risk: insights from vascular calcification biology and sclerostin signaling

**DOI:** 10.3389/fendo.2026.1839111

**Published:** 2026-04-29

**Authors:** Shuwei Weng, Chen Ding, Yuhong Shi, Hailin Zhang, Wenxiang Zhao, Jiang Zhu, Feng Peng, Dajun Chai

**Affiliations:** 1 Cardiovascular Department, The First Affiliated Hospital, Fujian Medical University, Key Laboratory of Metabolic Heart Disease in Fujian Province, Clinical Research Centre of Metabolic Cardiovascular Disease in Fujian Province, Fuzhou, China; 2 Cardiovascular Department, National Regional Medical Center, Binhai Branch of the First Affiliated Hospital, Fujian Medical University, Fuzhou, China

**Keywords:** cardiovascular safety, coronary artery calcification, osteoporosis therapies, romosozumab, vascular calcification

## Abstract

Osteoporosis therapies have become increasingly relevant to cardiovascular medicine because the bone–vascular interface links skeletal remodeling to vascular calcification biology. This narrative review examines whether anti-osteoporosis therapies truly modify coronary biology or risk, or whether the current literature more often reflects heterogeneous vascular end points, indirect coronary inference, and incomplete translation from mechanistic plausibility to clinical outcomes. We summarize the biologic pathways connecting bone remodeling to vascular calcification, including osteogenic transdifferentiation of vascular smooth muscle cells, matrix vesicle-mediated mineralization, the OPG/RANKL axis, and Wnt–sclerostin signaling. We then reassess current evidence for antiresorptive and osteoanabolic therapies while distinguishing coronary-specific data from broader vascular, renal-mineral, pharmacovigilance, and genetic evidence. Bisphosphonates and denosumab remain biologically relevant to vascular calcification, but based on currently available evidence, current studies do not support a reproducible or clinically decisive effect on coronary-specific outcomes. By contrast, romosozumab has sharpened the debate because it combines robust anti-fracture efficacy with unresolved cardiovascular safety questions linked to sclerostin inhibition, whose mechanistic, genetic, and clinical signals are not fully concordant. We further discuss why the literature remains conflicted, emphasizing end point heterogeneity, differences between calcification burden and plaque vulnerability, population-specific mineral stress, and the limitations of fracture trials for coronary inference. Overall, current evidence does not support a uniform cardiovascular class effect of osteoporosis therapies; instead, vascular consequences appear drug-specific, context-dependent, and highly sensitive to phenotype, baseline risk, and time horizon.

## 1 Introduction

Osteoporosis and coronary artery disease (CAD) are among the most prevalent chronic disorders of aging, and their coexistence is increasingly recognized in routine clinical practice rather than treated as a coincidental overlap (1). Over the last decade, observational studies and meta-analyses have repeatedly suggested that low bone mineral density (BMD) tracks with both angiographic CAD and coronary artery calcification (CAC), particularly in postmenopausal women; however, these associations often weaken after adjustment for age and cardiometabolic confounders, raising an immediate interpretive problem. The central question is no longer whether poor bone health and coronary disease can be observed in the same patient, but whether they are biologically connected, epidemiologically entangled, or both (2, 3).

That question matters because the older “bone loss versus vascular calcium gain” narrative is now too simplistic. Vascular calcification is not merely a passive precipitation of minerals in damaged tissue; it is an active, highly regulated process that shares striking molecular and cellular features with skeletal mineralization, including osteogenic reprogramming of vascular smooth muscle cells, matrix vesicle release, and the local balance between calcification promoters and inhibitors (4, 5). Within the coronary circulation, CAC is particularly important because it is not only a histopathologic expression of atherosclerotic burden but also a clinically useful imaging phenotype that refines cardiovascular risk prediction and increasingly influences preventive decision-making (6). These developments have shifted the field away from asking whether bone and coronary disease coexist, toward asking how disturbances in bone remodeling may intersect with coronary calcification biology.

Several signaling systems support the concept of a true bone–vascular axis. Osteoprotegerin (OPG), classically understood as a decoy receptor in bone remodeling, has long been linked to the presence and severity of CAD, while later work showed that elevated OPG also predicts cardiovascular morbidity and mortality in patients undergoing coronary assessment (7, 8). At the same time, pathways centered on Wnt signaling and its modulators, especially sclerostin, have drawn attention because they couple osteoblast biology to vascular smooth muscle cell transdifferentiation and calcific remodeling (9). These observations do not yet prove that bone-derived signals directly drive coronary events, but they do make it increasingly difficult to view osteoporosis and CAD as fully separate disease domains.

This mechanistic convergence has made anti-osteoporosis therapy a cardiovascular question. If bone-targeted drugs alter mineral handling, osteoclast–osteoblast signaling, or Wnt activity, then they may also influence vascular calcification or coronary risk. Yet the clinical evidence remains unsettled. Earlier meta-analytic work suggested that bisphosphonates might reduce arterial wall calcification without clearly reducing cardiovascular events, while a recent systematic review concluded that current evidence is still insufficient to establish whether bisphosphonates or denosumab meaningfully slow CAC progression (10, 11). Denosumab, despite a biologically plausible RANKL-mediated vascular effect, has not shown a consistent cardiovascular hazard signal across randomized data sets (12). Romosozumab has made the debate even sharper: by inhibiting sclerostin, it sits directly at the interface of bone anabolism and vascular biology, and although recent syntheses confirm robust skeletal efficacy, cardiovascular safety signals have not been fully resolved (13).

**Figure 1:**
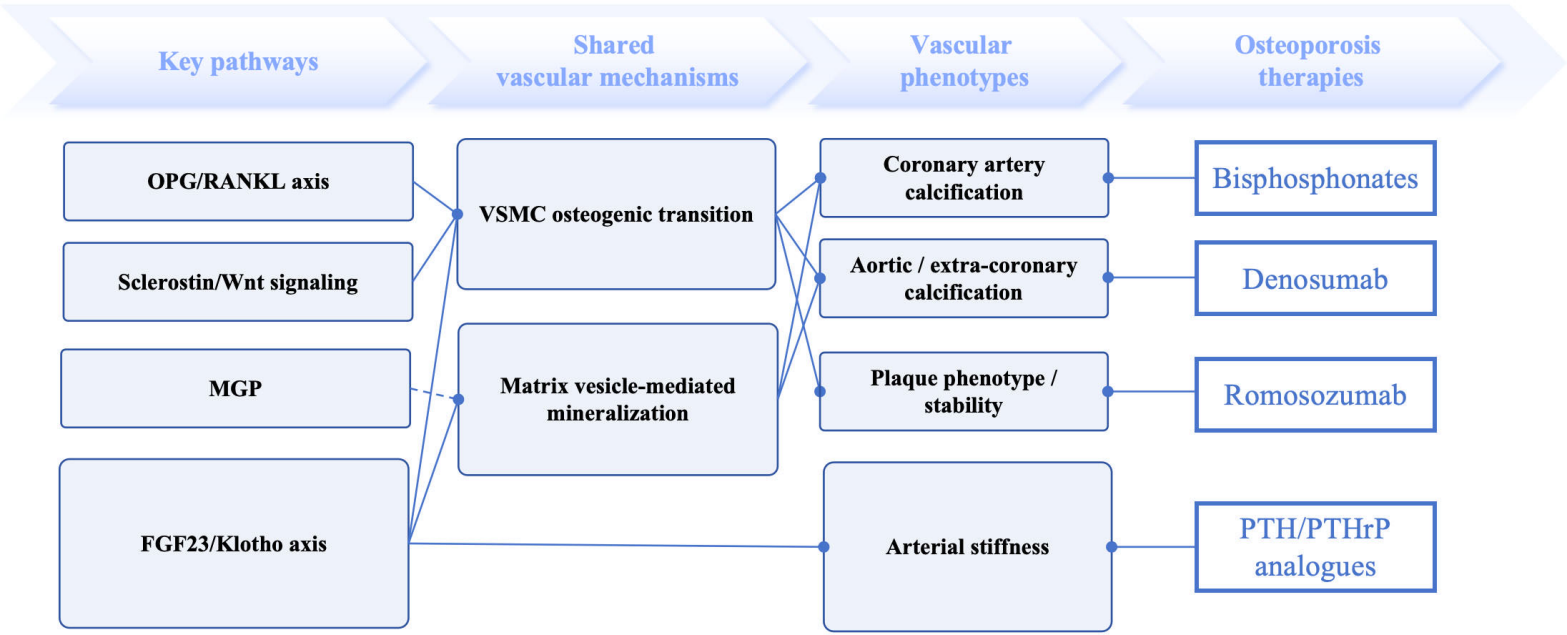
Conceptual framework linking bone–vascular pathways, shared vascular mechanisms, vascular phenotypes, and osteoporosis therapies relevant to coronary inference. Major bone–vascular pathways may converge on shared vascular mechanisms and contribute to distinct vascular phenotypes, including coronary artery calcification, aortic or extra-coronary calcification, plaque phenotype/stability, and arterial stiffness. The right panel links representative osteoporosis therapies to the vascular phenotypes for which relevant mechanistic or clinical evidence is currently available, highlighting that these relationships are drug-specific rather than uniform across therapies. Solid lines indicate mechanistic or conceptual links, whereas the dashed line indicates an inhibitory relationship, as illustrated for MGP. OPG, osteoprotegerin; RANKL, receptor activator of nuclear factor-κB ligand; MGP, matrix Gla protein; FGF23, fibroblast growth factor 23; VSMC, vascular smooth muscle cell; PTH, parathyroid hormone; PTHrP, parathyroid hormone-related peptide.

Against this background, the present review examines how osteoporosis therapies intersect with coronary calcification biology and cardiovascular risk. Rather than assuming a uniform cardiovascular class effect, we assess antiresorptive and osteoanabolic agents according to drug mechanism, vascular phenotype, and the strength of coronary-specific evidence. Particular attention is paid to the distinction between coronary imaging markers, broader vascular calcification phenotypes, and hard cardiovascular outcomes, because failure to separate these end points has contributed substantially to the current controversy (**Figure 1**).

This article was conducted as a narrative review. We searched PubMed/MEDLINE, Embase, and Web of Science for English-language studies from database inception to March 2026, and the search was updated during revision to incorporate newly published studies where relevant. The search combined terms related to osteoporosis therapies, including antiresorptive and osteoanabolic agents, as well as bone–vascular interactions, vascular calcification, coronary artery calcification, and cardiovascular outcomes. Representative keywords included osteoporosis, bisphosphonates, denosumab, romosozumab, teriparatide, abaloparatide, vascular calcification, coronary artery calcification, coronary artery disease, and sclerostin. We prioritized clinical trials, cohort studies, systematic reviews, and meta-analyses, while also incorporating mechanistic and preclinical studies when directly relevant.

## 2 The bone–vascular interface and its relevance to coronary calcification

The conceptual basis of a bone–vascular interface begins with the recognition that vascular calcification is an active, cell-regulated process rather than a passive sedimentation of calcium salts. Although much of the mechanistic evidence is not coronary-specific, these pathways remain relevant because coronary calcification emerges from broader vascular mineralization programs. Across experimental systems, vascular smooth muscle cells (VSMCs) emerge as the dominant cellular source of this osteogenic conversion. Under pro-calcific conditions, they progressively lose the contractile phenotype and acquire osteoblast-like features, with induction of transcriptional programs centered on RUNX2, alkaline phosphatase, and other bone-related genes (4, 5, 14, 15). This phenotypic switch matters for coronary disease because coronary artery calcification is not simply a radiographic footprint of prior injury; it is also the macroscopic product of a regulated mineralization program that shares machinery with skeletal remodeling.

A second key interface lies in the extracellular compartment. Calcifying VSMCs do not merely change their transcriptional identity; they also release matrix vesicles that serve as nucleation sites for hydroxyapatite deposition. These vesicles concentrate phosphatases, calcium-binding molecules, and membrane lipids that favor crystal formation, and they can propagate calcification from already injured or calcified cells to neighboring vascular cells (5, 16–18). In mechanistic terms, this is one of the clearest parallels between bone and artery: matrix vesicle–driven mineral initiation is fundamental to skeletal mineralization and appears to be repurposed in ectopic vascular calcification. Within coronary arteries, this biology offers a plausible bridge between systemic disturbances in bone turnover and the local progression of calcific plaque remodeling.

The balance between calcification promoters and inhibitors further reinforces this shared axis. Among the best-studied pathways is the OPG/RANKL system. In bone, OPG restrains osteoclastogenesis by acting as a decoy receptor for RANKL; in vessels, the same axis has been implicated in inflammatory activation, osteogenic signaling, and calcific remodeling (7, 8, 19). Importantly, this pathway illustrates one of the field’s enduring paradoxes: although OPG is often viewed as a protective factor in experimental calcification biology, higher circulating OPG in humans is commonly associated with more severe coronary disease and worse cardiovascular outcomes (8, 19). A similar complexity is seen with matrix Gla protein (MGP), a vitamin K-dependent inhibitor of vascular calcification produced by vascular cells. Functional MGP appears to oppose mineral deposition, yet clinical studies show that its circulating fractions behave heterogeneously across vascular beds and disease states, indicating that inhibition of calcification is context-dependent rather than uniform (20).

Wnt signaling provides another major mechanistic bridge between bone remodeling and coronary calcification. Canonical Wnt/β-catenin activity is central to osteoblast differentiation, and mounting evidence indicates that it also contributes to the osteochondrogenic transition of VSMCs during vascular calcification (9, 15, 21). Experimental studies further indicate that Wnt activation promotes matrix remodeling and calcific transformation, partly through induction of matrix metalloproteinases and osteogenic transcriptional programs (21). As an endogenous inhibitor of Wnt signaling, sclerostin is therefore particularly relevant to the present review because it lies at the interface between skeletal anabolism and vascular mineralization. Recent mechanistic work suggests that local vascular SOST/sclerostin is upregulated under calcifying stress, especially in chronic kidney disease (CKD)-associated vascular calcification, where it may represent a compensatory response to ongoing mineral stress rather than a simple pro-calcific driver (22). This pathway should not be treated as a single form of cardiovascular evidence. Three levels need to be distinguished: local vascular expression of sclerostin under calcifying stress, circulating sclerostin measured in clinical cohorts, and pharmacologic sclerostin inhibition with romosozumab. These levels are related, but they are not interchangeable. Increased vascular or circulating sclerostin may reflect a compensatory response to ongoing mineral stress rather than a simple pro-calcific signal. By contrast, therapeutic inhibition addresses a different question: whether blocking this pathway alters vascular biology or cardiovascular outcomes in treated patients. This separation helps explain why mechanistic plausibility does not necessarily translate into clinical event data (9, 22).

Finally, the bone–vascular interface becomes even more relevant under conditions of systemic mineral stress. Hyperphosphatemia, altered calcium handling, and disturbances in the FGF23/Klotho axis are particularly relevant in CKD, but the broader principle extends beyond nephrology: when the arterial wall is exposed to a systemic environment that favors ectopic mineral deposition and weakens endogenous inhibitory systems, vascular calcification intensifies and arterial stiffening becomes more pronounced (20, 23, 24). Coronary calcification should therefore not be viewed solely as a local plaque phenomenon, but also as a manifestation of whole-body mineral metabolism, endocrine signaling, and vascular susceptibility converging on the coronary bed. For the purposes of this review, this convergence is what makes osteoporosis therapies biologically plausible modifiers of coronary calcification, even when clinical outcome data remain inconsistent. A related biologic parallel is fracture repair. Although fracture healing and vascular calcification are not equivalent processes, both depend on tightly regulated inflammatory signaling, matrix remodeling, and context-dependent mineralization. This does not imply that vascular calcification is a reparative process in the skeletal sense; rather, it reinforces the broader concept that ectopic vascular mineralization and skeletal remodeling can share partial cellular machinery while diverging substantially in tissue context, biologic purpose, and clinical consequence (4, 5, 15–18, 23). Representative biomarkers involved in the bone-vascular pathway and their relevance to coronary calcification are summarized in **Table 1**.

**Table 1:** Representative biomarkers in the bone–vascular pathway and their relevance to coronary calcification.

Biomarker/pathway	Bone role	Vascular relevance	Key message	Key references
OPG/RANKL	Core regulators of osteoclastogenesis and bone resorption.	Linked to inflammatory signaling, osteogenic remodeling, and CAD severity.	A central bone–vascular axis, but circulating OPG should not be simplistically interpreted as protective in coronary disease.	(7, 8, 19)
MGP	Regulates mineralization balance.	Endogenous inhibitor of vascular calcification.	Best viewed as an anti-calcific marker, although its clinical behavior varies across vascular beds and disease states.	(20)
Sclerostin/Wnt	Inhibits Wnt signaling and suppresses bone formation.	Involved in VSMC osteogenic transition and CKD-associated vascular calcification.	Mechanistically important, but local vascular expression, circulating levels, and pharmacologic inhibition are not interchangeable.	(9, 22)
FGF23/Klotho	Controls phosphate and mineral metabolism.	Reflects systemic mineral stress that promotes vascular calcification, especially in CKD.	More useful as a context-dependent mineral-stress axis than as a direct coronary-specific marker.	(23, 24)

CAD, coronary artery disease; CKD, chronic kidney disease; MGP, matrix Gla protein; OPG, osteoprotegerin; RANKL, receptor activator of nuclear factor-κB ligand; VSMC, vascular smooth muscle cell.

## 3 Antiresorptive therapies: coronary-specific versus extra-coronary vascular evidence

Antiresorptive therapies provide one of the most informative pharmacologic tests of the bone–vascular hypothesis, although their coronary relevance must often be inferred rather than directly demonstrated. Because much of the available evidence comes from extra-coronary calcification studies, dialysis cohorts, or broader cardiovascular end points, these therapies should be interpreted as indirect probes of coronary biology rather than direct coronary interventions. If coronary calcification partly reflects a systemic mineralization phenotype, then suppressing skeletal resorption, altering calcium-phosphate flux, or modulating the OPG/RANKL system could plausibly reshape vascular calcification trajectories. At the same time, the expected direction of effect is not self-evident. Reduced bone turnover may limit mineral release and dampen osteogenic signaling, but it may also leave established vascular hydroxyapatite biologically untouched. This tension helps explain why antiresorptive therapies have generated persistent mechanistic optimism yet only mixed cardiovascular data (10, 11, 19). In this section, the evidence is interpreted by explicitly separating coronary-specific studies, particularly those evaluating coronary artery calcification, from studies based on aortic, extra-coronary, or dialysis-associated vascular calcification, because these end points are biologically related but not interchangeable for coronary inference (**Table 2**).

**Table 2:** Drug-class summary of coronary-specific versus extra-coronary vascular evidence.

Drug class	Coronary-specific evidence (CAC)	Extra-coronary vascular evidence	Overall cardiovascular signal	Take-home message	References
Bisphosphonates	No consistent effect on CAC progression; one recent population study suggested higher CAC.	Mixed findings across arterial wall and abdominal aortic calcification studies.	No reproducible cardiovascular benefit signal.	Coronary inference remains inconclusive; non-coronary data should not be overinterpreted as coronary evidence.	(10, 11, 25–28)
Denosumab	No consistent effect on CAC progression.	Mostly neutral overall; possible benefit in dialysis-associated or aortic calcification settings.	No consistent cardiovascular hazard signal.	Appears largely cardiovascularly neutral, with possible context-dependent effects in mineral-stress states.	(11, 12, 29–31)
Romosozumab	No direct CAC progression evidence cited.	No convincing human evidence for a consistent extra-coronary anti-calcific effect.	Cardiovascular safety signal remains debated across trials, real-world studies, pharmacovigilance, and genetic analyses.	The main unresolved cardiovascular controversy is concentrated in romosozumab, not in osteoporosis therapy as a whole.	(32–41)
PTH/PTHrP analogues	No direct CAC progression evidence cited.	No robust extra-coronary calcification signal identified.	No persistent major cardiovascular safety concern identified.	Current evidence appears more reassuring than for romosozumab, although direct coronary data are lacking.	(37, 38, 42)

CAC, coronary artery calcification; PTH, parathyroid hormone; PTHrP, parathyroid hormone-related peptide.

Bisphosphonates were the first osteoporosis drugs to attract serious cardiovascular interest, but their coronary-specific evidence should be considered separately from broader vascular calcification data because these layers of evidence do not support the same level of inference. In principle, they may interfere with hydroxyapatite crystal growth, influence macrophage activity, and attenuate osteogenic conversion within the vascular wall. However, coronary-specific evidence remains limited and inconclusive. A recent systematic review focused specifically on coronary artery calcification concluded that current evidence remains insufficient to support a clear association between bisphosphonate use and CAC progression (11). More recently, the Rotterdam Study reported that bisphosphonate use in the general population was associated with increased coronary artery calcification, with a duration-related pattern (25). Taken together, the available CAC-specific data do not support a stable coronary conclusion in either a protective or a harmful direction.

By contrast, extra-coronary evidence is broader but should not be interpreted as equivalent to CAC-based inference. Earlier clinical syntheses supported a possible vascular effect only partially: one systematic review and meta-analysis found that bisphosphonates were associated with reduced arterial wall calcification without a corresponding reduction in cardiovascular events (10), and a later clinical review suggested that any apparent benefit was more convincing in selected dialysis-related or extra-coronary calcification settings than in the broader osteoporosis population (26). Consistent with this uncertainty, once-yearly zoledronic acid did not slow the progression of abdominal aortic calcification over 3 years in the HORIZON Pivotal Fracture Trial analysis (27). A 2025 meta-analysis integrating animal and human studies likewise reported that nitrogen-containing bisphosphonates were not effective against vascular calcification in human studies, even though animal experiments suggested inhibition under some conditions (28). These extra-coronary data are biologically informative, but they do not resolve the coronary question directly.

Denosumab introduces a different biologic logic because it targets RANKL directly. Given the longstanding implication of the OPG/RANKL axis in vascular calcification biology, denosumab is arguably more mechanistically connected to the vessel wall than bisphosphonates are (19). This has made it an especially attractive candidate for testing whether antiresorptive therapy can alter vascular mineralization. However, coronary-specific evidence remains sparse and does not currently support a consistent effect on CAC progression. In the CAC-focused systematic review, current data did not support a consistent effect of RANKL inhibition on CAC progression (11). At the broader clinical level, randomized-trial evidence has likewise not shown a consistent overall cardiovascular safety signal across denosumab indications, supporting an interpretation closer to cardiovascular neutrality than to clear benefit or harm (12).

Extra-coronary evidence again appears more extensive than direct coronary evidence. The key *post hoc* analysis from the FREEDOM program reported that RANKL inhibition with denosumab did not influence 3-year progression of aortic calcification or the incidence of adverse cardiovascular events in postmenopausal women with osteoporosis and high cardiovascular risk (29). A 2024 meta-analysis found no definite inhibitory effect of denosumab on vascular calcification overall, but suggested a more favorable effect in patients with CKD undergoing dialysis (30). Supporting this possibility, an observational study in long-term hemodialysis patients reported that denosumab was associated with reduced aortic arch calcification over 30 months, whereas calcification progressed in controls (31). Accordingly, denosumab may be best understood not as a universally anti-calcific drug, but as one whose vascular effects become more visible in mineral-driven disease states such as advanced CKD; importantly, these findings arise mainly from aortic or dialysis-associated settings rather than direct CAC studies.

From a coronary perspective, the current antiresorptive evidence supports a restrained interpretation. For both bisphosphonates and denosumab, CAC-specific evidence remains limited and does not demonstrate a reproducible or clinically decisive coronary effect. By contrast, aortic, extra-coronary, and dialysis-associated calcification studies provide biologically informative but non-equivalent evidence. These vascular beds and patient contexts should therefore not be collapsed into a single coronary narrative. This distinction is essential when antiresorptive data are used to support broader claims about coronary biology or cardiovascular risk (10–12, 19, 29–31).

## 4 Osteoanabolic therapies, sclerostin inhibition, and cardiovascular concern

Osteoanabolic therapy raises a different cardiovascular question from antiresorptive treatment because it does not simply suppress skeletal turnover; rather, it actively shifts bone remodeling toward formation. Among currently available anabolic agents, romosozumab occupies a uniquely sensitive position in the bone–vascular conversation because it targets sclerostin, a regulator of Wnt signaling that has already been implicated in vascular calcification biology (9, 22). Clinically, romosozumab has shown impressive anti-fracture efficacy. In the placebo-controlled FRAME trial, romosozumab markedly reduced vertebral and clinical fractures, and cardiovascular events appeared balanced between treatment groups (32). In contrast, in the active-comparator ARCH trial, romosozumab followed by alendronate reduced vertebral, clinical, nonvertebral, and hip fractures more effectively than alendronate alone, but positively adjudicated serious cardiovascular adverse events during the first year were numerically more frequent in the romosozumab group (33). It was this asymmetry between efficacy and safety, and between trials with different comparators, that transformed romosozumab from a straightforward bone anabolic therapy into a broader cardiovascular controversy (32, 33).

Subsequent evidence has narrowed the extremes of interpretation without fully dissolving the concern. In a 2024 real-world cohort study, romosozumab was not associated with an increase in major adverse cardiovascular events during treatment, although absolute event rates were higher in patients with pre-existing cardiovascular disease or diabetes (34). A placebo-controlled meta-analysis published the same year found no significant increase in cardiovascular adverse events within 12 months, while a 2025 network meta-analysis of randomized trials likewise found no significant difference in cardiovascular mortality or major cardiovascular events overall and further suggested that no significant excess cardiovascular event signal was observed when romosozumab was compared with active comparators other than alendronate (35, 36). Still, these syntheses should not be over-read. Cardiovascular events were relatively infrequent, follow-up was limited, and the signal that generated the greatest concern emerged in a head-to-head comparison against alendronate rather than placebo. For that reason, the most defensible interpretation is not that romosozumab has been fully exonerated, but that the available trial-level evidence is insufficient to prove a consistent excess risk across all patient populations.

Why, then, does concern persist? Part of the answer lies beyond randomized trial summaries. A pharmacovigilance analysis of FAERS detected disproportionate reporting of major adverse cardiovascular events with romosozumab, supporting regulatory caution even though spontaneous reporting systems cannot establish causality (40). More recent real-world evidence has been more nuanced. In a propensity-score-matched real-world cohort study, romosozumab was associated with fewer 3-point major adverse cardiovascular events over 1 year than parathyroid hormone analogues, suggesting that the cardiovascular signal may not be uniformly unfavorable across comparative clinical settings (37). A population-based cohort study comparing romosozumab with teriparatide similarly found no significant difference in MACE between the two drugs over 365 days (38). At the same time, a 2024 cis-Mendelian randomization study reported that genetically predicted lower sclerostin levels were associated not only with higher bone density and lower hip fracture risk, but also with excess coronary artery disease risk and adverse cardiometabolic biomarker profiles (39). This genetic signal does not prove that romosozumab itself causes coronary events, but it helps explain why the cardiovascular debate around sclerostin inhibition continues even when conventional meta-analyses appear reassuring (37–40).

These apparently divergent signals are best understood as different evidentiary layers rather than direct contradictions. Randomized trials ask whether therapeutic sclerostin inhibition increases short- to medium-term cardiovascular events in selected osteoporosis populations. Pharmacovigilance analyses test whether safety signals emerge in broader clinical use, but remain vulnerable to reporting bias and channeling. Mendelian randomization addresses a different question again, namely whether lifelong genetically lower sclerostin is linked to coronary risk and adverse cardiometabolic traits. None of these designs is individually definitive, but together they suggest that sclerostin signaling remains biologically relevant to coronary disease even though the magnitude, timing, and clinical expression of any drug-related hazard remain unresolved (35–40).

By contrast, the broader osteoanabolic class has not generated an equally persistent coronary safety controversy. PTH or PTHrP-based anabolic agents, such as teriparatide and abaloparatide, do not directly target the sclerostin–Wnt axis and therefore occupy a different biologic space. In the ACTIVE phase 3 analysis, abaloparatide was associated with transient increases in heart rate and small decreases in blood pressure, but not with increases in serious cardiac adverse events, MACE, or heart failure (42). Meanwhile, nonclinical cardiovascular studies of romosozumab failed to identify functional, morphologic, or transcriptional evidence that sclerostin inhibition accelerates atheroprogression or cardiovascular calcification in animal models, underscoring the ongoing gap between mechanistic plausibility and clinical signal detection (41). Taken together, the available data suggest that cardiovascular concern in osteoanabolic therapy is not a simple class effect. It is instead concentrated around romosozumab, shaped by the biologic specificity of sclerostin inhibition, and sustained by a mismatch between highly persuasive fracture efficacy and still-incomplete certainty regarding cardiovascular safety (41, 42).

Taken together, the current therapeutic picture is uneven rather than uniformly reassuring or uniformly harmful. For bisphosphonates and denosumab, the dominant signal is biologic plausibility without reproducible coronary benefit or a consistent cardiovascular hazard. For romosozumab, the dominant signal is a more clinically consequential tension between robust skeletal efficacy and unresolved cardiovascular safety. For other osteoanabolic agents, available evidence remains comparatively limited and has not generated a similarly persistent coronary controversy. Framed this way, the major challenge is no longer whether osteoporosis therapies have any vascular relevance, but how strongly that relevance can be inferred for each drug class and in which patient context.

## 5 Why the evidence remains conflicted

The persistent inconsistency in this field does not necessarily mean that the bone–coronary hypothesis is false; rather, it reflects the fact that much of the available evidence is not truly coronary-specific, even when it is used to support coronary interpretations. Coronary artery calcification, extra-coronary arterial calcification, aortic arch calcification, angiographic coronary stenosis, and hard cardiovascular events are frequently discussed within the same evidentiary frame, even though they are not interchangeable outcomes. This matters for drug interpretation. A therapy could alter mineral deposition within the vessel wall without measurably changing plaque burden. It could also influence plaque composition without generating a detectable signal in major adverse cardiovascular events over the limited follow-up typical of osteoporosis trials (10–12, 29–36). Serial CAC is an imperfect therapeutic endpoint for the same reason. Its progression is influenced by baseline calcium burden, scan reproducibility, and the mathematical properties of calcium scoring, all of which complicate the inference that a change in CAC trajectory necessarily represents a clinically meaningful change in coronary risk (43).

A second source of conflict is that calcification burden does not map neatly onto plaque instability. Coronary calcium is a robust marker of atherosclerotic burden and future cardiovascular risk at the patient level. However, plaque vulnerability is influenced not only by total calcium burden, but also by calcification morphology and distribution. In the Multi-Ethnic Study of Atherosclerosis, calcium density was inversely associated with incident cardiovascular events after adjustment for plaque volume, suggesting that denser calcification may reflect a more stable phenotype than less consolidated calcium (44). A recent systematic review and meta-analysis reached a similar conclusion, showing that higher CAC density is associated with lower cardiovascular event risk (45). Pathobiology and imaging studies are consistent with this distinction. Microcalcification and spotty superficial calcium may be linked to plaque vulnerability, whereas larger sheet-like or densely mineralized calcification may represent a later and potentially more stable stage of lesion evolution (46, 47). For this reason, a drug-related increase, decrease, or neutral effect on total calcification burden cannot automatically be interpreted as an increase, decrease, or neutral effect on coronary event risk (44–47).

Population heterogeneity adds a third layer of complexity. The biology of vascular calcification in advanced CKD differs substantially from that in typical postmenopausal osteoporosis cohorts. In CKD, calcification is more tightly linked to systemic mineral stress, phosphate retention, altered calcium handling, and endocrine disturbances involving PTH, vitamin D, FGF23, and Klotho; moreover, medial calcification becomes more prominent alongside intimal atherosclerotic calcification, and this process is commonly accompanied by increased arterial stiffness and loss of vascular compliance (23, 24, 48, 49). This helps explain why denosumab or bisphosphonate signals may appear more favorable in dialysis-associated calcification studies than in general osteoporosis populations (26, 28, 30, 31). It also means that evidence generated in high-mineral-stress settings cannot be casually extrapolated to coronary risk in community-dwelling women with osteoporosis. When studies with such different biological substrates are synthesized together, apparent contradiction may simply reflect unrecognized context dependence (23, 24, 48, 49).

Study design further amplifies the problem. Most randomized osteoporosis trials were designed around fracture efficacy rather than cardiovascular adjudication. As a result, event counts are relatively low and follow-up windows are often too short to clarify whether a vascular signal is real, absent, or delayed (32–36, 40). Comparator choice also matters. The cardiovascular concern surrounding romosozumab was amplified by the ARCH trial, in which the comparator was alendronate rather than placebo (33). That design cannot fully distinguish whether the observed imbalance reflects an adverse effect of sclerostin inhibition, a protective effect of alendronate in a high-risk population, random variation, or some combination of these explanations (33–36, 40, 50).

Observational studies create a different interpretive problem. They provide longer follow-up and broader clinical exposure, but they are intrinsically vulnerable to confounding by indication. Patients selected for osteoporosis treatment are often older, frailer, and more comorbid than untreated comparators. In addition, the choice between agents is itself influenced by baseline cardiovascular history, renal function, fracture risk, and clinician caution. Under these conditions, both apparent harm and apparent safety can partly reflect allocation patterns rather than pure drug biology (28, 37, 38, 50). Taken together, the conflicting literature is best understood not as a failure of the field, but as the consequence of collapsing different levels of biology, imaging, and clinical inference into a single narrative. The key unresolved issue is therefore not simply whether osteoporosis therapies affect “the cardiovascular system,” but which vascular phenotype, in which patient population, over what time horizon, and by what mechanism. Until studies align drug exposure with calcification phenotype, coronary-specific imaging, baseline cardiovascular risk, and adequately powered clinical outcomes, disagreement will remain structurally built into the evidence base.

The present review should also be interpreted in light of several limitations. It is a narrative synthesis of heterogeneous evidence rather than a formal systematic review, and the literature itself remains uneven in coronary specificity, endpoint definition, and causal interpretability. For that reason, our aim is not to offer definitive cardiovascular adjudication for osteoporosis therapies, but to clarify how different evidentiary layers should be weighed in coronary interpretation.

## 6 Clinical considerations for interpretation

Clinical interpretation may be most appropriately anchored in fracture urgency rather than in the expectation that an osteoporosis drug will independently improve coronary outcomes. Current guidance and expert review place romosozumab primarily in the setting of very high fracture risk, where rapid and substantial skeletal benefit is needed, and subsequent sequencing with a potent antiresorptive agent is part of the intended strategy rather than an optional afterthought. In practical terms, this means that patients with multiple recent vertebral fractures, extremely low bone mineral density, or failure of prior antiresorptive therapy are those in whom the anabolic advantage of romosozumab is most clinically relevant. By contrast, when fracture urgency is lower, it becomes harder to justify accepting even an unresolved cardiovascular signal for the sake of incremental skeletal gain alone, particularly when romosozumab is being considered outside the broader context of planned sequential therapy (51–53).

An additional clinical consideration is whether sequential antiresorptive therapy may modify cardiovascular interpretation after romosozumab. In routine practice, romosozumab is generally used as an initial anabolic phase and is then followed by a potent antiresorptive agent to consolidate skeletal gains rather than continued as a long-term standalone strategy (51–53). This treatment sequence complicates cardiovascular inference. The imbalance in positively adjudicated serious cardiovascular adverse events in ARCH emerged during the first year of romosozumab exposure against alendronate (33), whereas more recent real-world comparative studies have not shown a consistent excess of major cardiovascular events across broader clinical settings (34, 37, 38). Although these data do not prove that subsequent antiresorptive therapy neutralizes a short-term vascular signal, they do suggest that the imbalance observed in a single trial setting should not be assumed to represent a stable or uniformly persistent long-term cardiovascular hazard after transition to routine sequential care. For that reason, cardiovascular interpretation after romosozumab should be based not only on the initial anabolic phase, but also on the full therapeutic sequence, the subsequent antiresorptive phase, and the patient’s baseline cardiovascular risk.

A further interpretive distinction is to separate established or very high cardiovascular risk from the broader and far more common background of age-related risk factors. The Endocrine Society guideline update emphasizes that romosozumab carries a boxed warning and requires careful consideration of the individual cardiovascular risk profile (51). More recent expert pieces have moved the field toward a more explicit pre-treatment assessment model. Macrae and colleagues argue that cardiovascular risk scoring should be incorporated into osteoporosis practice (54). A recent review likewise emphasizes that treatment decisions in patients at high cardiovascular risk should weigh skeletal benefit against potential cardiovascular harm rather than treating the issue as already settled (55). Accordingly, a cautious interpretation is that romosozumab may be less attractive in patients with clearly established recent atherothrombotic disease or very high cardiovascular risk, and may be better reserved for situations in which fracture risk is sufficiently urgent to justify individualized benefit–risk discussion.

In patients who do not have overt recent cardiovascular disease, cardiovascular risk factors should not be treated as automatic exclusion criteria, but neither should they be ignored. Real-world and review data suggest that any excess event signal is more likely to cluster in patients with pre-existing cardiovascular disease, diabetes, or other high-risk profiles than to emerge uniformly across all treated patients (34, 39). This supports a more refined interpretive approach in which hypertension, diabetes, smoking, lipid disorders, and prior vascular history are explicitly considered and, where appropriate, optimized before therapy is initiated. Mild-to-moderate CKD also deserves nuance. *Post hoc* analyses from FRAME and ARCH indicate that romosozumab preserved efficacy and had an acceptable overall safety profile in women with mild-to-moderate CKD, so reduced kidney function in that range should not automatically preclude treatment (56). However, because advanced CKD is a biologically distinct calcification state driven by mineral stress, extrapolation from general osteoporosis populations should remain cautious (23, 24, 56).

## 7 Future directions

Future research will likely need to move beyond asking whether osteoporosis therapies change “calcification” in a generic sense and instead define which coronary phenotype is being modified. Coronary calcium burden, calcium density, spotty calcification, plaque composition, and active microcalcification are biologically related but clinically non-equivalent measures. Recent work on CAC density progression has reinforced that density itself may convey information distinct from total calcium volume, while newer evidence continues to support the inverse relationship between CAC density and cardiovascular events (43–45, 57). Accordingly, future trials would be better served by not relying solely on Agatston score progression. Coronary imaging phenotypes that distinguish plaque burden from plaque biology should ideally be prespecified, because they are more closely aligned with the mechanisms through which bone-targeted therapies may act.

A second priority is to incorporate coronary-specific multimodality imaging into mechanistic and translational studies. Coronary CT angiography can characterize high-risk plaque features beyond calcified burden alone, and ^18F-sodium fluoride PET provides a complementary window into active microcalcification rather than established macroscopic calcium (46, 47, 58–60). These tools are especially important for this field because a therapy might reduce active calcification, alter plaque phenotype, or modify the transition from microcalcification to dense calcification without generating an obvious short-term signal in total CAC score. Future studies would be strengthened by pairing treatment exposure with baseline and follow-up coronary phenotyping, ideally integrating CAC, CCTA plaque characterization, and, where feasible, PET-based microcalcification activity. Such an approach would allow investigators to test more directly whether bone-active drugs influence coronary plaque biology rather than merely shifting a crude imaging surrogate.

Third, the field needs better causal designs rather than simply more observational comparisons. Randomized fracture trials remain essential, but most are underpowered for cardiovascular end points and often lack coronary-specific imaging. Observational studies can contribute valuable real-world evidence, but only if they are designed to minimize confounding by indication, immortal time bias, and comparator imbalance. The target trial emulation framework has emerged precisely to improve causal inference from observational data, and it is particularly relevant here because treatment selection in osteoporosis is strongly shaped by fracture severity, renal function, cardiovascular history, and clinician risk aversion (50, 61). In parallel, Mendelian randomization should be used more strategically, not as a substitute for clinical evidence, but as a way to test whether mechanistic targets such as sclerostin are likely to have on-target coronary consequences before those questions are left to *post hoc* pharmacovigilance alone (39, 62). The most informative future evidence base will likely require triangulation across randomized data, target trial emulations, and genetically anchored causal inference rather than reliance on any single design.

Finally, future studies will likely need to be explicitly population-stratified. Advanced CKD, dialysis, severe baseline CAC, established CAD, and very high fracture risk are unlikely to be nuisance variables alone; they are more plausibly effect modifiers. The next generation of research should ask not only whether a given drug is “cardiovascularly safe,” but also in whom, over what time horizon, and against which baseline coronary substrate. Clinically, this would support a more useful framework in which osteoporosis therapies are interpreted through joint skeletal and coronary phenotyping rather than through isolated drug labels. Scientifically, it would move the field away from the false binary of benefit versus harm and toward a more precise model of context-dependent vascular response (23, 24, 39, 48, 49, 57–62).

## 8 Conclusion

In summary, current evidence does not support a uniform cardiovascular class effect of osteoporosis therapies. Instead, antiresorptive and osteoanabolic agents appear to interact with coronary biology in heterogeneous, context-dependent ways that are likely shaped by drug mechanism, baseline vascular substrate, mineral metabolic milieu, and patient-level cardiovascular risk. In particular, sclerostin signaling should be interpreted across distinct evidentiary layers, including vascular expression, circulating biomarkers, genetic proxies, and pharmacologic inhibition, rather than reduced to a single harmful-versus-protective label. Bisphosphonates and denosumab remain biologically relevant to vascular calcification but have not shown a consistent or clinically decisive ability to alter coronary-specific outcomes or their validated surrogates, whereas romosozumab has brought the bone–vascular axis into sharper clinical focus by combining strong skeletal efficacy with a cardiovascular safety debate that remains unresolved rather than settled. The central challenge for the field is therefore not simply to determine whether bone-targeted therapies are “good” or “bad” for the heart, but to define which coronary phenotypes they may influence, in which patients, and over what time horizon.
